# ZAR1/2‐Regulated Epigenetic Modifications are Essential for Age‐Associated Oocyte Quality Maintenance and Zygotic Activation

**DOI:** 10.1002/advs.202410305

**Published:** 2025-01-04

**Authors:** Yan Rong, Yu‐Ke Wu, Yingyan Chen, Qing Liu, Leilei Ai, Yun‐Wen Wu, Yezhang Zhu, Yin‐Li Zhang, Chengkan Liu, Yerong Ma, Xiaomei Tong, Jiamin Jin, Xiaoxuan Li, Yan Zhou, Shu‐Yan Ji, Songying Zhang, Heng‐Yu Fan

**Affiliations:** ^1^ Department of Obstetrics and Gynecology Zhejiang Key Laboratory of Precise Protection and Promotion of Fertility Zhejiang Provincial Clinical Research Center for Reproductive Health and Disease Assisted Reproduction Unit Sir Run Run Shaw Hospital School of Medicine Zhejiang University Hangzhou 310016 China; ^2^ MOE Key Laboratory for Biosystems Homeostasis and Protection and Innovation Center for Cell Signaling Network, Life Sciences Institute Zhejiang University Hangzhou 310058 China; ^3^ Department of Traditional Chinese Medicine Sir Run Run Shaw Hospital Zhejiang University School of Medicine Hangzhou 310016 China; ^4^ Center for Biomedical Research Shaoxing Institute Zhejiang University Shaoxing 312000 China

**Keywords:** histone modifications, maternal factor, oocyte epigenetic maturation, oocyte‐to‐embryo transition, reproductive aging

## Abstract

The developmental competence and epigenetic progression of oocytes gradually become dysregulated with increasing maternal age. However, the mechanisms underlying age‐related epigenetic regulation in oocytes remain poorly understood. Zygote arrest proteins 1 and 2 (ZAR1/2) are two maternal factors with partially redundant roles in maintaining oocyte quality, mainly known by regulating mRNA stability. In addition to this known function, it is found that ZAR1/2 is required for oocyte epigenetic maturation and zygotic reprogramming. *Zar1/2*‐deleted oocytes exhibited reduced levels of multiple histone modifications and of the expression of corresponding histone modifiers, along with over‐condensed chromatin, leading to compromised minor zygotic genome activation and deficient embryo development following fertilization. Cytoplasmic ZAR1/2 participated in intranuclear epigenetic maturation by binding the transcripts encoding histone modifiers and regulating their stability and translational activity. Moreover, oocytes from aged mice exhibited similar histone‐modification deficiencies as the *Zar1/2*‐deleted oocytes. ZAR1/2 mRNA and protein levels are downregulated in oocytes from mice and women with advanced ages, suggesting ZAR1/2 as regulators of epigenetic changes with reproductive aging. This study presents a new nucleo‐cytoplasmic interaction mechanism that is involved in preventing oocyte epigenetic aging. Further, *ZAR1/2* represents potential gene targets for diagnosis and clinical interventions in age‐associated deficiencies in oocyte and embryo development.

## Graphical abstract

Cytoplasmic ZAR1/2 participates in intranuclear epigenetic maturation by regulating the stability and translation of the transcripts encoding histone modifiers. In the absence of ZAR1/2, oocytes exhibit reduced levels of multiple histone modifications and modifiers, along with over‐condensed chromatin, subsequently leading to compromised minor ZGA. It is further proved that ZAR1/2 as regulators play vital roles in preventing oocyte epigenetic aging.

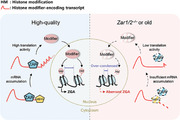

## 1 Introduction

Despite human longevity has increased with the development of modern society and medical technology, the female reproductive system ages decades before other organ systems. Reproductive aging, characterized by oocyte quality decline and endocrine environment changes, occurs when females reach their mid‐thirties and carries increased risks of embryo lethality, miscarriage, infertility, twinning, and congenital birth defects,^[^ ^1^, ^2^, ^3^ ^]^ ultimately leading to the cessation of reproductive function at menopause.^[^ ^4^ ^]^ Oocytes in older women exhibit many deleterious effects, including increased chromosomal aneuploidy, impaired maternal mRNA accumulation, translation and decay, mitochondrial dysfunction, zonal hardening, telomere shortening, and epigenetic changes.^[^ ^5^, ^6^, ^7^, ^8^, ^9^, ^10^ ^]^ Moreover, data from assisted reproductive cycles shows that older women who conceive using eggs from young donors get better fertility outcomes,^[^ ^11^ ^]^ suggesting that low‐quality oocytes, rather than defects in the other reproductive organs, present a common and insuperable handicap for women with advanced age, being the principal cause of poor reproductive outcomes. As many women choose to postpone childbearing because of lifestyle changes and economic pressures, reproductive aging is becoming a worldwide societal problem.

The integration of transient or long‐term accumulated environmental signals on chromatin states alters oocyte gene expression and downstream cellular events.^[^ ^12^ ^]^ In recent years, oocyte quality has been studied by evaluating the establishment and maintenance of epigenetic modifications, owing to their persistence and progressive changes with age.^[^ ^13^ ^]^ The mechanisms and factors that sustain oocyte epigenetic maturation with age require further exploration.

Zygote arrest‐1 (ZAR1) and ZAR2 (also known as ZAR1L) are two key maternal factors. Their C‐terminal CxxC zinc finger domains are highly conserved RNA‐binding structures in vertebrates, despite the fact that ZAR proteins have divergent spatiotemporal expression among species.^[^ ^14^, ^15^ ^]^ In *Xenopus laevis* and zebrafish, ZAR proteins bind maternal transcripts to inhibit their translation in immature oocytes.^[^ ^16^, ^17^ ^]^ In mice, ZAR proteins stabilize maternal transcripts in growing oocytes and promote their translation during meiosis, and maternal ZAR1/2 deficiency leads to zygotic arrest.^[^ ^18^ ^]^ In all, ZAR1 and ZAR2 (ZAR1/2), which are to some extent functionally redundant, are necessary for oocyte quality control and maternal‐to‐zygotic transition (MZT).

In mouse zygotes, ZAR2 C‐terminus overexpression leads to embryonic arrest at the 2‐cell stage; H3K4me2/3 and H3K9me2 are markedly down‐regulated in arrested embryos, whereas H3K4me1 and H3K9me3 are up‐regulated.^[^ ^19^ ^]^ Correspondingly, individual chromatin modification components, such as *Dppa2*, *Dppa4*, and *Piwil2*, and global transcription activity are significantly down‐regulated, suggesting that ZAR2 participates in methylation‐associated histone modification to ensure zygotic genome activation (ZGA).^[^ ^19^, ^20^ ^]^ MSY2, another RNA‐binding protein and a component of the ZAR1‐mediated mRNA accumulation compartment mitochondria‐associated ribonucleoprotein domain (MARDO),^[^ ^21^ ^]^ also participates in histone modification. *Msy2*‐deleted oocytes exhibited substantial reductions in H3K9me3, H3K4me3, and H4K5/8/12/16ac modifications.^[^ ^22^ ^]^ We have previously found that *Zar1/2*‐deleted oocytes exhibit reduced MSY2 expression.^[^ ^18^ ^]^ However, it remains unclear whether ZAR proteins affect histone modifications in vivo.

Oocytes are ideal models for studying chromatin modifications, because transcriptional silencing and reprogramming respectively occur in fully grown oocytes and after fertilization, where de novo epigenetic modification is necessary.^[^ ^23^ ^]^ During normal gametogenesis and development, histone modification is ongoing and serves important functions. With maternal aging, oocytes exhibit epigenetic defects and changes in chromatin configuration.^[^ ^8^, ^13^, ^24^, ^25^ ^]^

Here, we found that *Zar1/2*‐deleted oocytes exhibited decreased histone modification levels and over‐condensed chromatin, similar to old oocytes. Reduced levels of transcripts and proteins involved in histone modifications were also observed in maternal ZAR1/2‐deficient oocytes/zygotes and oocytes from older individuals. Changes in histone modification and chromatin state contributed to zygotic activation failure in the zygotes exhibiting maternal ZAR1/2 deficiency. Moreover, ZAR1/2 levels were reduced in oocytes from old women and mice. These findings reveal a network by which cytoplastic ZAR1/2 regulate epigenetic modifications in the nucleus and play vital roles in age‐associated oocyte and early embryo quality maintenance.

## 2 Results

### 2.1 ZAR1/2‐Deletion Impairs Histone Modification in Oocytes

**Figure 1:**
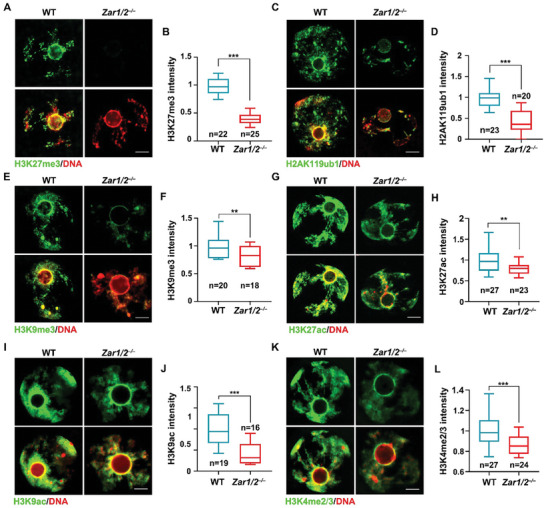
ZAR1/2‐deletion impaired histone modifications in GV stage‐arrested mouse oocytes. A,C,E,G,I,K) Immunofluorescence of H3K27me3 (A), H2AK119ub1 (C), H3K9me3 (E), H3K27ac (G), H3K9ac (I), and H3K4me2/3 (K) in WT and *Zar1/2^–/–^* oocytes. Scale bar, 5 µm. B,D,F,H,J,L) Quantification of histone modification signal intensity of (A,C,E,G,I,K), respectively. Error bars, SEM.

To explore the effect of ZAR1/2 on histone modifications in oocytes, we used *Zar1* and *Zar2* double knockout mouse model (*Zar1/2^–/–^*) which were reported previously.^[^ ^18^ ^]^ Although ZAR1 and ZAR2 are cytoplasmic proteins, multiple histone modifications changed within the oocyte nucleus after their deletion. *Zar1/2^–/–^* oocytes exhibited reduced levels of H3K27me3 (**Figure** **1**A,B), H2AK119ub1 (Figure 1C,D), and H3K9me3 (Figure 1E,F) modifications, related to transcriptional inhibition, and of H3K27ac (Figure 1G,H), H3K9ac (Figure 1I,J), H3K4me2/3 (Figure 1K,L) and H4K5/8/12/16ac (Figure S1A,B, Supporting Information) modifications, related to transcriptional activation, with some of these modifications occurring at the same sites (such as H3K27me3 and H3K27ac at one site and H3K9me3 and H3K9ac at another). Levels of the H3K9me3, H3K4me3, and H4K5/8/12/16ac modifications are also reduced in oocytes lacking MSY2, a partner of ZAR1 to promote MARDO assembly.^[^ ^18^, ^21^, ^22^ ^]^ Considering that ZAR1/2 deletion leads to reduced MSY2 levels,^[^ ^18^ ^]^ we suggest that the ZAR1‐associated MARDO complex possibly contributes to epigenetic modification in oocytes.

### 2.2 ZAR1/2‐Deletion Impairs mRNA Accumulation and Translation of Histone Modifiers in Oocytes

**Figure 2:**
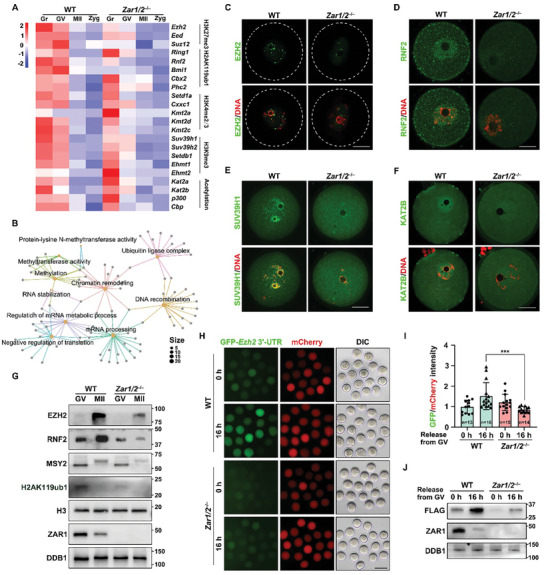
ZAR1/2‐deletion impaired mRNA and protein accumulations of histone modifiers during mouse oocyte development. A) Heat map illustrating differences in the levels of the representative histone modifier‐encoding transcripts in oocytes and zygotes from WT and *Zar1/2^–/–^* females. B) GO analysis of transcripts significantly down‐regulated in *Zar1/2^–/–^* GV oocytes. C–F) Immunofluorescence of EZH2 (C), RNF2 (D), SUV39H1 (E), and KAT2B (F) in GV oocytes derived from WT and *Zar1/2^–/–^* females. Scale bar, 20 µm. G) Western blotting illustrating the levels of indicated proteins in GV and MII oocytes of WT and *Zar1/2^–/–^* females. DDB1 was used as the loading control. H) Fluorescence microscopy illustrating the expression of FLAG‐GFP fused with the *Ezh2* 3′‐UTR in WT and *Zar1/2^−/−^* oocytes. Scale bar, 100 µm. I,J) Relative GFP intensity (I) and western blotting (J) results illustrate the expression of FLAG‐GFP fused with the *Ezh2* 3′‐UTR in WT and *Zar1/2^−/−^* oocytes. Error bars, SEM. DDB1 was used as the loading control.

We have previously reported that ZAR1/2 deletion led to maternal mRNA instability.^[^ ^18^ ^]^ To examine why these histone modifications were decreased in the absence of ZAR1/2, we first analyzed transcription‐level changes of the relevant histone modifiers in oocytes. RNA‐seq revealed that, in WT oocytes, various transcripts encoding histone modification‐related proteins were maintained at relatively high levels during oocyte growth and were downregulated following meiotic maturation (**Figure** **2**A), as most maternal transcripts did. However, these transcripts (such as *Ezh2*, *Rnf2*, *Suv39h1*, et al.) were expressed at significantly lower levels in growing *Zar1/2^–/–^* oocytes than in WT oocytes, and this effect became more apparent at the fully grown stage (Figure 2A; Figure S2A, Supporting Information). Modifiers that erased histone modifications exhibited a similar down‐regulation (Figure S2B, Supporting Information), suggesting that the low level of histone modifications was caused by the failure to establishment rather than by over‐removal. Gene Ontology (GO) analysis revealed that the down‐regulated transcripts in *Zar1/2^–/–^* GV oocytes were enriched in mRNA processing, translation, and mRNA metabolism, as well as in methylation, ubiquitylation, and chromatin remodeling (Figure 2B), consistent with the observed ZAR1/2‐associated oocyte defects. These suggest that inadequate accumulation of histone modification‐related transcripts, owing to the lack of ZAR1/2, possibly contributes to these defects.

The protein levels of these histone modifiers were also examined. Levels of the H3K27 methyltransferase EZH2,^[^ ^26^, ^27^ ^]^ the ubiquitin ligase RNF2,^[^ ^28^ ^]^ the H3K9 methyltransferase SUV39H1^[^ ^29^ ^]^ and the acetyltransferase KAT2B^[^ ^30^ ^]^ were all reduced following ZAR1/2 deletion (Figure 2C–F; Figure S2C–F, Supporting Information). Western blotting also revealed lower levels of EZH2, RNF2, and H2AK119ub1 in *Zar1/2^–/–^* oocytes (Figure 2G). MSY2 was reduced in *Zar1/2^–/–^* oocytes, consistent with our previous findings.^[^ ^18^ ^]^ Levels of histone H3, the eukaryotic nucleosome octamer core component, were not affected by ZAR1/2 deletion. This indicates that these histone modification disorders are caused by reductions in modifier levels rather than in chromatin histone levels (Figure 2G). We tried to supplement mCherry‐RNF2 in *Zar1/2^–/–^* oocytes but the H2AK119ub1 level could not be rescued (Figure S2G,H, Supporting Information). Indeed, PRC1/2 complex had many components, and just supplementing one or two components is unlikely to rescue the situation.

We also checked the epigenetic states of *Zar1/2*‐knockout MII oocytes. As reported, H2AK119ub1 and multiple histone acetylation were removed in MII oocytes.^[^ ^31^, ^32^ ^]^ *Zar1/2^–/–^* MII oocytes also exhibited low H2AK119ub1, H3K27ac, H3K9ac, and H4K5/8/12/16ac levels, again proving that the erasing of histone modifications was not significantly impaired after ZAR1/2 deletion. Besides, under an extremely high‐intensity exposure, these modifications of WT MII oocytes could show signals. However, H2AK119ub1, H3K27ac, H3K9ac, and H4K5/8/12/16ac levels of *Zar1/2^–/–^* MII oocytes were more deceased than those in WT MII oocytes (Figure S2I,J, Supporting Information), which was similar to that at GV stage. Besides, H3K27me3, H3K9me3 and H3K4me2/3 were also impaired in *Zar1/2^–/–^* MII oocytes (Figure S2I,J, Supporting Information).

In addition to the reduced mRNA levels, whether the compromised translation also contributed to the histone modification disorders remained to be evaluated. We next examined the contribution of compromised translation to the reduction in histone modifier levels by constructing FLAG‐GFP reporters fused to the 3′‐untranslated region (3′‐UTR) of mouse *Ezh2*. Following in vitro transcription into mRNAs without polyadenylation, reporter transcripts were microinjected into WT and *Zar1/2^–/–^* oocytes at the GV stage. In vitro, polyadenylated *mCherry* transcripts were co‐injected as a positive control. After 16 h of mRNA microinjection, GFP signals were detected in the GV‐ and MII‐arrested oocytes. In WT oocytes, the GFP signals were stronger at the MII stage than at the GV stage, as was EZH2 level in Figure 2G, suggesting that *Ezh2* 3′‐UTR could drive the meiotic maturation‐coupled translation of this reporter. In *Zar1/2^–/–^* oocytes, the signals were markedly reduced, whereas the mCherry signals were equal in each group (Figure 2H,I). Similar results were obtained using Western blotting (Figure 2J). These results indicate that the translational efficiencies of the modifiers also contribute to the ZAR1/2‐regulated establishment of histone modifications.

### 2.3 Maternal ZAR1/2 Contributes to Proper Reprogramming of Zygotic Histone Modifications After Fertilization

Fertilization begins with the fusion of two specialized gametes, followed by major epigenetic remodeling mediated by maternal factors stored in the cytoplasm, and ultimately the formation of a totipotent embryo. Although levels of H3K27me3 and H2AK119ub1 did not increase during oocyte maturation,^[^ ^32^, ^33^ ^]^ their modification enzymes, EZH2 and RNF2, accumulated significantly (Figure 2G), suggesting that pre‐accumulated maternal modifiers could be involved in epigenetic remodeling after fertilization. Precise reprogramming is a prerequisite for establishing embryo totipotency,^[^ ^12^ ^]^ however, inadequate RNF2 and EZH2 in *Zar1/2*‐knockout oocytes at the MII stage implies that these oocytes may exhibit insufficient histone modification after fertilization.

**Figure 3:**
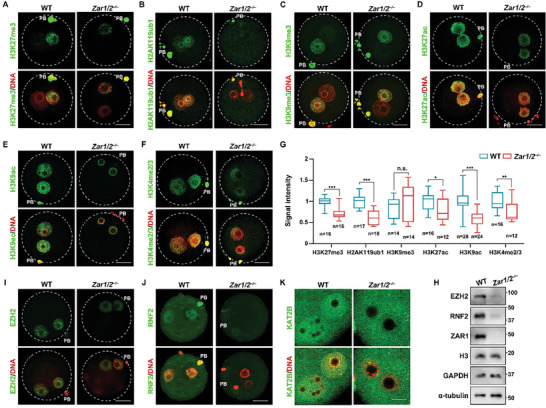
Maternal ZAR1/2 contributed to proper zygotic histone modifications. A–F) Immunofluorescence of H3K27me3 (A), H2AK119ub1 (B), H3K9me3 (C), H3K27ac (D), H3K9ac (E), and H3K4me2/3 (F) in zygotes from WT and *Zar1/2^–/–^* females. Scale bar, 20 µm. G) Quantification of histone modification signal intensity of (A–F). PB, polar body. Error bars, SEM. I–K) Immunofluorescence of EZH2 (I), RNF2 (J), and intranuclear KAT2B (K) in zygotes from WT and *Zar1/2^–/–^* females. Scale bar, 20 µm in (I–J) and 5 µm in (K). H) Western blotting illustrating the levels of indicated proteins in zygotes from WT and *Zar1/2^–/–^* females. GAPDH and α‐tubulin were used as the loading controls.

To validate this, we mated superovulated WT and *Zar1/2^–/–^* females with WT males and collected the zygotes at 28 h post‐hCG injection. Immunofluorescence imaging revealed that maternal *Zar1/2*‐deleted zygotes (*Zar1/2^♀–/♂+^*) exhibited significantly reduced levels of H3K27me3, H2AK119ub1, H3K27ac, H3K9ac, and H3K4me2/3 but not H3K9me3 and H4K5/8/12/16ac modifications (**Figure** **3**A–G; Figure S1C,D, Supporting Information). As expected, the levels of their corresponding modification enzymes, including EZH2, RNF2, and KAT2B, were significantly reduced following maternal ZAR1/2 deletion at the zygote stage (Figure 3I–K), although their transcript levels were not significantly altered (Figure 2A). This was also validated by Western blotting (Figure 3H). In conclusion, maternal ZAR1/2 may contribute to histone modifications both in oocytes and in zygotes.

### 2.4 ZAR1/2 Interact with Maternal Transcripts Encoding Histone Modifiers

**Figure 4:**
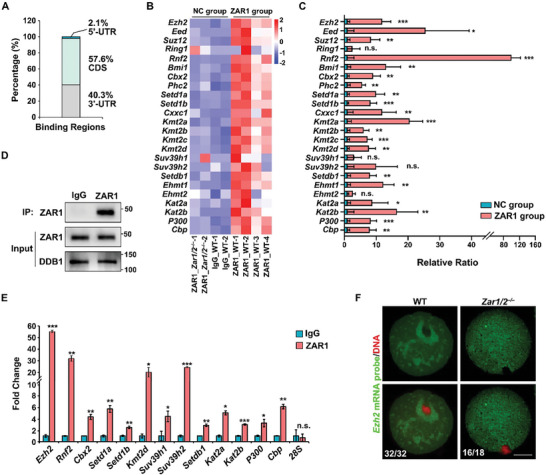
ZAR1 interacted with maternal mRNAs encoding histone modifiers to regulate epigenetic modifications in mouse oocytes. A) Bar graph showing the distribution of ZAR1 binding sites. B) LACE‐seq illustrating changes in the levels of the representative histone modifier‐encoding transcripts bound by the anti‐ZAR1 antibody. The left two columns, the third and fourth left columns, and the four right columns represented the transcripts bound by anti‐ZAR1 antibody in *Zar1/2^–/–^* oocytes, by anti‐IgG antibody in WT oocytes, and by anti‐ZAR1 antibody in WT oocytes, respectively. C) LACE‐seq illustrating the relative ratios of the transcripts from the NC and ZAR1 groups. Error bars, SEM. D) Western blotting illustrating the endogenous ZAR1 protein pulled down from WT oocyte lysates by the anti‐ZAR1 antibody. E) RIP assay results illustrating the interactions between ZAR1 and the histone modifier‐encoding transcripts in GV oocytes. Levels of transcripts coprecipitated with ZAR1 were detected by RT‐qPCR. *n* = 3 biological replicates. Error bars, SEM. F) The FISH assay shows the distribution of *Ezh2* mRNAs in WT and *Zar1/2^−/−^* oocytes. Scale bar, 20 µm.

The C‐termini of ZAR1/2 containing RNA‐binding domains are evolutionarily conserved among vertebrates. To investigate whether ZAR1/2 regulate the stability and translation of transcripts encoding histone modifiers by directly interacting with them, we collected WT and *Zar1/2^–/–^* oocytes for Linear amplification of cDNA ends and sequencing (LACE‐seq). *Zar1/2^–/–^* and WT oocytes with IgG antibody were used as negative controls (NC) groups. We confirmed that ZAR1 targeted the transcripts on their 3′‐UTR (40.3%) and CDS (57.6%) regions (**Figure** **4**A). Besides, most of the histone modifier‐related transcripts list in Figure 2A were enriched in ZAR1 precipitates of WT oocytes (Figure 4B,C).

RNA immunoprecipitation (RIP) assays were also performed in WT oocytes. After endogenous ZAR1 proteins were specifically pulled down from oocyte lysates using an anti‐ZAR1 antibody (Figure 4D), RT‐qPCR was performed, revealing that maternal transcripts encoding proteins essential for histone modifications, including *Ezh2*, *Rnf2*, *Kat2b*, were enriched in ZAR1 precipitates (Figure 4E). RIP assays using 293T cells produced similar results: exogenous human ZAR1 (hZAR1) was overexpressed in 293T cells (Figure S3A, Supporting Information), which do not express endogenous ZAR1 proteins, and the selected transcripts were found to bind with hZAR1 based on RT‐qPCR (Figure S3B, Supporting Information).

We also detected the localization of ZAR1/2‐targeted transcripts using a series of Cy3‐labeled fluorescent probes that recognized *Ezh2* mRNAs (Table S2, Supporting Information). The results of FISH assays showed that *Ezh2* mRNAs were clustered around the spindle of WT oocytes and their distribution resembled MARDO. ZAR1/2 deletion destroyed this MARDO‐like distribution and made them uniform in the cytoplasm (Figure 4F). Therefore, ZAR1/2 interacts with histone modifier‐encoding mRNAs to potentially regulate their stability and translation, participating in establishing epigenetic modifications in oocytes.

### 2.5 Maternal ZAR1/2 Deletion‐Mediated Histone Modification Disorders Lead to Chromatin Tightening

**Figure 5:**
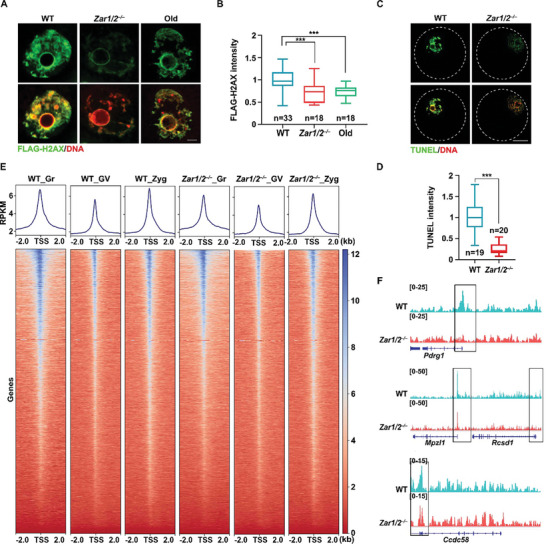
Effect of maternal ZAR1/2 deletion on chromatin accessibility. A) Immunofluorescence of FLAG illustrating the incorporation of FLAG‐histone H2AX in WT and *Zar1/2^–/–^* oocytes, as well as old oocytes. Scale bar, 5 µm. B) Quantification of chromatin‐incorporated FLAG‐histone H2AX signals in (A). Error bars, SEM. C) DNase I‐TUNEL assay on WT and *Zar1/2^–/–^* oocytes illustrating the accessibility of genomic DNA to DNase I. Scale bar, 20 µm. D) Quantification of TUNEL signal intensity of WT and *Zar1/2^–/–^* oocytes after DNase I digestion. Error bars, SEM. E: ATAC‐seq enrichment around accessible promoters identified in growing oocytes, GV oocytes, and zygotes from WT and *Zar1/2^–/–^* females. F) IGV view showing ATAC‐seq enrichment in zygotes from WT and *Zar1/2^–/–^* females. The black frames indicated corresponding promoter regions.

Histone post‐translational modification and modifier changes are closely related to specific chromatin states.^[^ ^34^, ^35^ ^]^ Owing to the marked defects in histone modification observed in *Zar1/2*‐deleted oocytes, we examined whether these defects also disrupted the chromatin state. The active exchange of histone variants (H2AX and H3.3) in oocyte chromatin is an indicator of chromatin tightness and accessibility.^[^ ^34^ ^]^ Therefore, we performed a histone replacement experiment by exogenously expressing FLAG‐tagged histone variants in WT and *Zar1/2^–/–^* oocytes. After 24 h of culture, the histone variants incorporated into the chromatin were detected by immunofluorescence using an anti‐FLAG antibody. The chromatin of *Zar1/2*‐deleted oocytes exhibited less incorporation of de novo synthesized H2AX and H3.3 than that of WT oocytes (**Figure** **5**A,B; Figure S4A,B, Supporting Information). We also incubated WT and *Zar1/2^–/–^* oocytes with DNase I and measured the DNA double‐strand break signal via a TUNEL assay. The genomic DNA of *Zar1/2^–/–^* oocytes was more resistant to DNase I digestion than that of the WT oocytes (Figure 5C,D). Besides, the chromatin accessibility of *Zar1/2^♀–/♂+^* zygotes was decreased as that of oocytes (Figure S4C,D, Supporting Information).

To further assess the effect of ZAR1/2 on chromatin accessibility, we collected growing oocytes, fully grown oocytes, and zygotes for an improved assay for transposase‐accessible chromatin with high‐throughput sequencing (ATAC‐seq). Indeed, growing oocytes had the highest chromatin accessibility, which was correlated with their active transcription. Zygotes had the second highest chromatin accessibility, suggesting that early embryos started to enable the zygotic transcription activities. The lowest chromatin accessibility was observed in fully grown oocytes which were at the transcriptional silencing state. Notably, all *Zar1/2^–/–^* groups had decreased ATAC‐seq enrichments around accessible promoters compared to their respective WT groups (Figure 5E,F). Thus, histone modification changes by maternal ZAR1/2 deletion may lead to increased chromatin tightness and reduced chromatin accessibility in oocytes and zygotes derived from these oocytes.

Although reduced levels of histone modifiers could be associated with increased chromatin tightness, chromatin condensation may in turn prevent histone modifiers from coming into contact with chromatin, thus leading to reduced epigenetic modifications. To verify this possibility, we overexpressed mCherry‐tagged RNF2 and EZH2 in WT and *Zar1/2^–/–^* oocytes via mRNA microinjections. Similar signal intensities were detected in oocytes of both genotypes 24 h after microinjections (Figure S4E–H, Supporting Information), suggesting that chromatin condensation did not influence the incorporation of histone modifiers. Therefore, multiple histone modification disorders are likely due to the reduced histone modifier levels.

### 2.6 Maternal Transcript Degradation and Minor ZGA are Impaired in Maternal *Zar1/2* Deleted Zygotes

In mouse embryos, initial ZGA is initiated at the late 1‐cell stage, which is known as minor ZGA, and major ZGA occurs at the 2‐cell stage.^[^ ^36^ ^]^ Previous studies have shown that over 90% of zygotes from *Zar1/2^–/–^* females arrest at the 1‐cell stage, and the remaining 10% arrest at the two‐cell stage and exhibit impaired major ZGA.^[^ ^18^ ^]^ Therefore, we investigated the effects of maternal‐derived epigenetic and chromatic disorders on transcription in maternal *Zar1/2* knockout zygotes.

**Figure 6:**
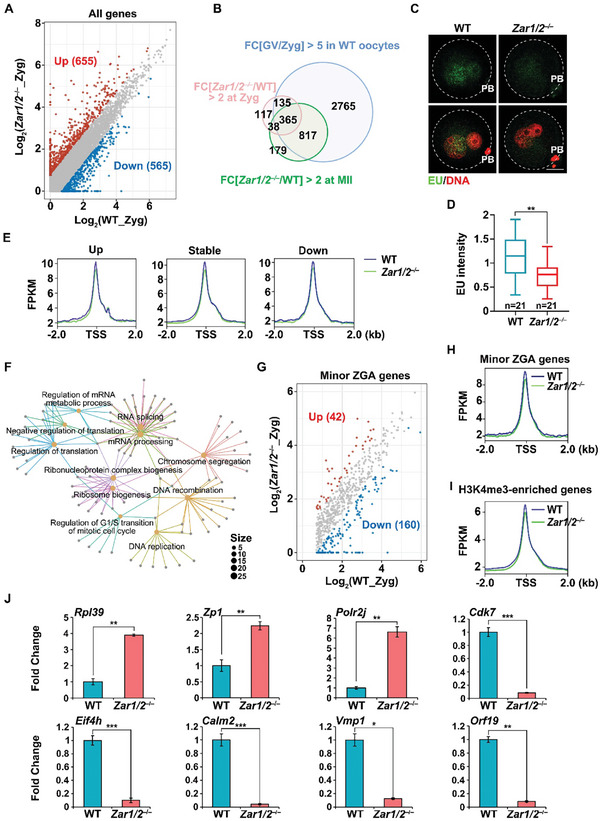
Maternal ZAR1/2 deletion disrupted minor ZGA in mice. A) Scatter plots of RNA‐seq data illustrating transcriptional changes in WT and maternal *Zar1/2*‐deleted zygotes. Transcripts >2‐fold up‐ or down‐regulated relative to the WT zygotes were highlighted in red and blue, respectively. B) Venn diagram illustrating the overlap of the transcripts that decreased from the GV to zygote stage in WT oocytes and transcripts that accumulated in MII oocytes and zygotes from *Zar1/2*‐deleted females. C) Detection of newly synthesized RNA via EU incorporation in zygotes from WT and *Zar1/2^–/–^* females. Scale bar, 20 µm. D) Quantification of EU signal intensity of zygotes from WT and *Zar1/2^–/–^* females. Error bars, SEM. E) ATAC‐seq enrichment around accessible promoters of up‐, not‐ and down‐regulated genes in (A) in zygotes from WT and *Zar1/2^–/–^* females. F) GO analysis of transcripts significantly down‐regulated in maternal *Zar1/2*‐deleted zygotes. G) Scatter plots of RNA‐seq data illustrating transcriptional changes of minor ZGA genes between WT and maternal *Zar1/2*‐deleted zygotes. H) ATAC‐seq enrichment around accessible promoters of minor ZGA genes identified in zygotes from WT and *Zar1/2^–/–^* females. I) ATAC‐seq enrichment around accessible promoters of H3K4me3‐enriched genes identified in zygotes from WT and *Zar1/2^–/–^* females. J) RT‐qPCR illustrating the relative levels of indicated transcripts in zygotes of WT and *Zar1/2^−/−^* females. *n* = 3 biological replicates. Error bars, SEM.

RNA‐seq of the zygotes revealed that 655 transcripts were up‐regulated [fold change (WT/ *Zar1/2^♀–/♂+^*) < 1/2] and 565 transcripts were down‐regulated [fold change (WT*/Zar1/2^♀–/♂+^*) > 2] after maternal ZAR1/2 deletion (**Figure** **6**A; Figure S5A, Supporting Information). Of the up‐regulated transcripts, 76.3% should have exhibited normal degradation before or after fertilization, 59.2% of which were accumulated in *Zar1/2^–/–^* oocytes at the MII stage (Figure 6B), and 63.2% were involved in BTG4‐dependent RNA decay pathway (Figure S5B, Supporting Information). Because ZAR1/2 participate in maternal mRNA degradation both before and after fertilization, these up‐regulated transcripts were associated with abnormal degradation instead of increased *de novo* transcription. Besides, the EU incorporation assay further proved that the overall genomic transcriptional activity of zygotes was substantially reduced following maternal ZAR1/2 deletion (Figure 6C,D), which was consistent with the abnormally tightened chromatin states (Figure 6E). All the genes, regardless of down‐regulated, unchanged, or up‐regulated, had a more pronounced decreased in ATAC‐seq enrichments in *Zar1/2^♀–/♂+^* zygotes (Figure 6E).

For the down‐regulated transcripts, GO analysis revealed that they were enriched in chromosome segregation and cell cycle control (Figure 6F), partially explaining the observed zygotic arrest. We further concentrated on the genes involved in minor ZGA and found that 160 minor ZGA genes were down‐regulated while 42 were up‐regulated in *Zar1/2^♀–/♂+^* zygotes (Figure 6G), further indicating that maternal ZAR1/2 deletion disrupted minor ZGA. Both minor ZGA genes and H3K4me3‐enriched genes (analyzed from published data^[^ ^37^ ^]^) showed decreased ATAC‐seq enrichments in promoter regions after maternal ZAR1/2 deletion (Figure 6H,I). However, the ATAC‐seq enrichments of histone modifier‐encoding genes were hardly affected (Figure S5C, Supporting Information), again suggesting that the decline of histone modifiers after ZAR1/2 deletion was due to decreased transcript stability and translation, rather than abnormal transcription. RT‐qPCR also verified that specific maternal transcripts which should have been degraded before the zygote stage, such as *Rpl39*, *Zp1*, and *Polr2j*, were maintained at high levels in the *Zar1/2^♀–/♂+^* zygotes. The minor transcriptional activation of the genes was blocked, including that of the cell cycle‐related gene *Cdk7* and translation‐related gene *Eif4* *h*, and that of other genes (*Calm2*, *Vmp1*, and *Orf19*) with no known functions in the MZT (Figure 6J). These indicate that maternal ZAR1/2 absence impairs maternal transcript degradation and minor ZGA, at least partially leading to embryonic development failure.

### 2.7 Potential Involvement of ZAR1/2‐Dependent Histone Modifications in Mouse and Human Oocyte Aging

*Zar1/2*‐deleted oocytes and old oocytes share many characteristics, including increased chromosomal aneuploidy, low translation activity, impaired maternal mRNA accumulation and degradation, and mitochondrial dysfunction,^[^ ^9^, ^18^, ^25^ ^]^ suggesting that ZAR1/2 could be vital regulators of oocyte aging. Recently, histone‐modification disorders in oocytes from older individuals have been reported.^[^ ^9^, ^24^, ^25^, ^38^ ^]^ However, no prior studies have suggested whether ZAR1/2 are associated with epigenetic modifications during oocyte aging.

**Figure 7:**
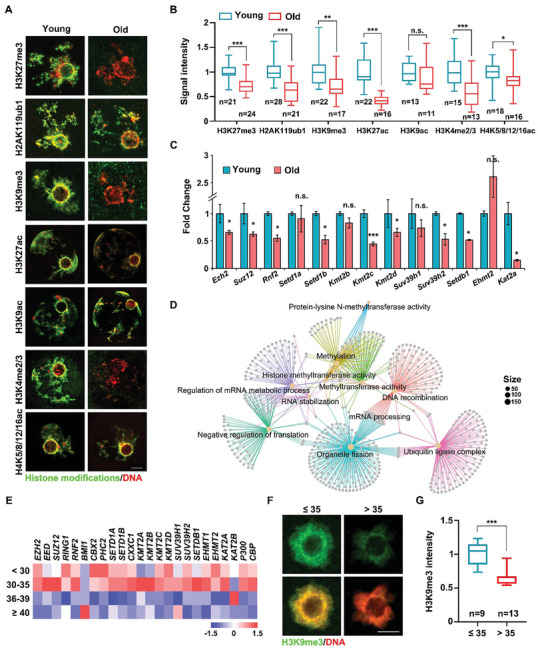
Age‐associated reductions of histone modifiers in mouse and human oocytes. A) Immunofluorescence of multiple histone modifications in oocytes from younger and older mice. Scale bar, 5 µm. B) Quantification of histone modification signal intensity of the histone modifications in (A). Error bars, SEM. C) RT‐qPCR illustrating relative levels of representative histone modifier‐encoding transcripts in oocytes from younger and older mice. *n* = 3 biological replicates. Error bars, SEM. D) GO analysis of transcripts significantly down‐regulated (>2‐fold) in GV oocytes from women ≥ 40 years old, compared with oocytes from women < 30 years old. E) Heat map illustrating the changes in the expression of histone modifier‐encoding transcripts in human GV oocytes of different ages. F) Immunofluorescence of H3K9me3 in oocytes from women ≤ 35 and > 35 years old. Scale bar, 5 µm. G) Quantification of H3K9me3 signal intensity in (F). Error bars, SEM.

**Figure 8:**
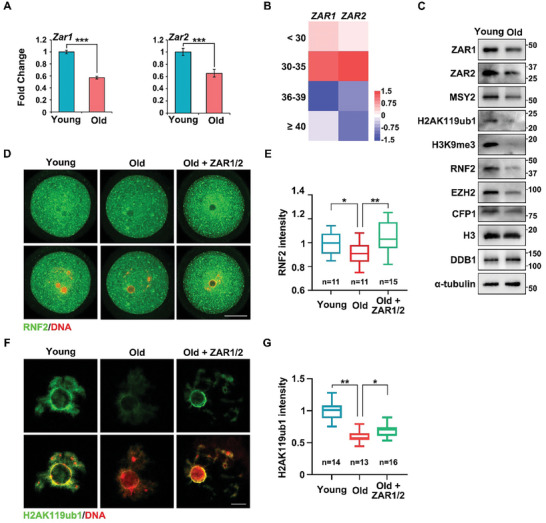
Potential involvement of ZAR1/2‐mediated histone modifications in oocyte aging. A) RT‐qPCR revealed the relative levels of *Zar1* and *Zar2* in oocytes from younger and older mice. *n* = 3 biological replicates. Error bars, SEM. B) Heat map illustrating differences of *ZAR1* and *ZAR2* levels in GV oocytes from women of different ages. C) Western blotting illustrating protein levels in oocytes from younger and older mice. α‐tubulin and DDB1 were used as the loading control. D,F) Immunofluorescence illustrating RNF2 (D) and H2AK119ub1 (F) levels of oocytes from younger and older mice, as well as old oocytes overexpressing FLAG‐ZAR1 and FLAG‐ZAR2. Scale bar, 20 µm in (D) and 5 µm in (F). E,G) Quantification of RNF2 (D) and H2AK119ub1 (F) signal intensity. Error bars, SEM.

The levels of H3K27me3, H2AK119ub1, H3K9me3, H3K27ac, H3K9ac, H3K4me2/3, and H4K5/8/12/16ac modifications, already observed in *Zar1/2^–/–^* oocytes, were significantly reduced in oocytes derived from 14‐month‐old mice (**Figure** **7**A,B). Transcripts for the genes encoding these histone‐modifying enzymes, including *Ezh2*, *Rnf2*, *Setd1b*, *Suv39h2*, and *Kat2a*, were down‐regulated in oocytes from these older mice (Figure 7C), as were the corresponding protein levels (Figure 8C). The histone exchange assay revealed that less de novo‐synthesized H2AX was incorporated into the chromatin of oocytes from older individuals as into that of *Zar1/2^–/–^* oocytes (Figure 5A,B), reflecting the tightened chromatin state and reduced chromatin accessibility in the oocytes of older mice.

Next, we analyzed published RNA‐seq data for human GV oocytes.^[^ ^25^ ^]^ GO analysis suggested that the down‐regulated transcripts in the oocytes from older women [fold change (<30*/*≥45) >2] were associated with mRNA processing and metabolism, translation, and histone modifications such as methylation and ubiquitylation (Figure 7D), similar to the associations exhibited for the down‐regulated genes in *Zar1/2^–/–^* oocytes (Figure 2B). Most of the histone modifier‐encoding transcripts listed in Figure 2A were down‐regulated in the human oocytes with advancing age (Figure 7E). We also conducted the immunofluorescence assay on oocytes from young and old women who came to our reproductive center for assisted reproduction, following proper ethical regulations. H3K9me3 levels were decreased in oocytes from women with advanced ages (Figure 7F,G), which was similar to that in mice (Figure 1E,F). To a certain extent, the epigenetic degradation in old oocytes is conserved between humans and mice.

Moreover, both *Zar1* and *Zar2* were down‐regulated in the GV oocytes from older mice (**Figure** **8**A), with human oocytes exhibiting a similar pattern (Figure 8B). Western blotting revealed lower ZAR1/2 and MSY2 levels in oocytes from older mice than in those from younger mice (Figure 8C). To further identify the roles of ZAR1/2 in oocyte epigenetic aging, we constructed the plasmids overexpressing ZAR1 and ZAR2 (Figure S6A, Supporting Information), and performed rescue experiments: mRNA microinjection‐induced ectopic expression of FLAG‐ZAR1 and ‐ZAR2 in oocytes from older individuals partially improved the level of RNF2 and corresponding H2AK119ub1 (Figure 8D–G), which was closely related to ZGA. Unfortunately, EZH2, H3K27me3, and H3K27ac levels could not be rescued (Figure S6B–G, Supporting Information), indicating the complexity of epigenetic regulation. These results suggest a critical association between ZAR1/2‐mediated histone modification and oocyte aging.

We compared the epigenetic states of oocytes from 21‐day‐old and 4‐month‐old *Zar1/2^–/–^* females, and found that H3K27me3, but not H2AK119ub1 or H3K27ac, was decreased in the older oocytes (Figure S6H,I, Supporting Information). We further tried to supplement ZAR1 into *Zar1/2^–/–^* oocytes but failed to rescue H2AK119ub1 levels (Figure S6J,K, Supporting Information). *Zar1/2* of oocytes are highly expressed since the primordial follicle stage.^[^ ^18^ ^]^ The lack of transcripts encoding histone modifiers gradually emerges during the growth of ZAR1/2‐deleted oocytes. Just supplement of ZAR1 at the fully grown stage is too late because transcription has already been silenced. For this reason, the histone modification deficiency is more severe in the oocytes completely devoid of ZAR1/2 than in old oocytes.

## 3 Discussion

Oocyte quality is a key limiting factor for female fertility, but the drivers and mechanisms of oocyte quality remain poorly understood. Precise epigenetic modification and reprogramming during oocyte and early embryo development are prerequisites for avoiding developmental defects or embryonic lethality. Many factors play vital roles in these processes. Here, two well‐known maternal factors, ZAR1 and ZAR2, were found to be associated with histone modification and oocyte aging.

ZAR1 and ZAR2 are important for oocyte quality control and for MZT and exhibit partially functional redundancy. They are conserved in vertebrates, especially at their C‐termini.^[^ ^15^ ^]^ ZAR1 is first identified by Wu in 2003; in mice, its deletion leads to female infertility.^[^ ^14^ ^]^ In *Xenopus laevis*, ZAR1/2 bind to the translational control sequences in the 3′‐UTR of maternal mRNAs to repress their translation, with different characteristics.^[^ ^39^ ^]^ During early oogenesis in zebrafish, ZAR1 binds to zona pellucida transcripts and represses their translation; its deletion induces ER stress, apoptosis, and sex reversal.^[^ ^17^ ^]^ Two novel SNPs of ZAR1 are reported to be associated with human zygote arrest.^[^ ^40^ ^]^ Besides, ZAR1/2 are recognized as novel epigenetically inactivated tumor suppressors in various types of cancer.^[^ ^41^, ^42^, ^43^, ^44^, ^45^ ^]^ We have previously systematically examined the in vivo and redundant functions of ZAR proteins in regulating oocyte maturation and MZT by generating *Zar1/2* double knockout mice, revealing that ZAR1/2 bind to mRNAs to maintain maternal transcriptome stability by interacting with other proteins (such as MSY2 and cytoplasmic lattice components) and to promote translation activation in oocytes.^[^ ^18^ ^]^

Oocyte maturation involves nuclear, cytoplasmic, and epigenetic maturation.^[^ ^25^, ^46^ ^]^ The involvement of ZAR2 in epigenetic modification has been reported: in two‐cell stage arrested embryos that overexpressing the C‐terminus of ZAR2, levels of H3K4me2/3 and H3K9me2 are down‐regulated, while those of H3K9me3 are up‐regulated; further, the transcripts of chromatin modification components and the global transcriptional activity of these arrested embryos are significantly down‐regulated.^[^ ^19^ ^]^ Oocytes lacking MSY2, another component of MARDO, exhibit markedly reduced levels of H3K9me3, H3K4me3, and H4K5/8/12/16ac,^[^ ^22^ ^]^ although the underlying mechanisms remain unclear.

Here, we examined the mechanisms whereby cytoplasmic ZAR1/2 regulated epigenetic maturation. Levels of various histone modifications, including H3K27me3, H2AK119ub1, H3K9me3, H3K27ac, H3K9ac, H3K4me2/3, and H4K5/8/12/16ac, were reduced in maternal *Zar1/2*‐deleted oocytes and zygotes, leading to abnormal chromatin states and blocked minor ZGA. The novel perspectives on nucleo‐cytoplasmic interactions presented here will support future mechanistic studies.

Oocytes from older individuals exhibit similar deficiencies to *Zar1/2* knockout oocytes. The rate of chromosomal abnormalities increases with advancing age, owing to changes in chromosome structure, chromosome‐associated factors, spindle assembly checkpoint sensitivity, and cell cycle regulation.^[^ ^47^, ^48^, ^49^, ^50^ ^]^ In addition, disordered maternal mRNA accumulation and decay, reduced translation activity, and mitochondrial dysfunction are prominent hallmarks of oocyte aging.^[^ ^25^ ^]^ Besides nuclear and cytoplasmic defects described above, our study revealed that epigenetic defects also appear as an important phenotype in maternal *Zar1/2* knockout oocytes and zygotes.

The relationships between histone modification and aging have been examined in various species. During aging, changes in histone modifications may affect cellular function and stress resistance, and the regulation effects of different modifying enzymes on the same modification are usually not the same. In *Caenorhabditis elegans*, knockdown of UTX‐1, an H3K27me3 demethylase associated with transcriptional regulation, can prolong the lifespan, suggesting that H3K27me3 levels are positively correlated with lifespan.^[^ ^51^ ^]^ However, its lifespan can also be prolonged by the overexpression of another H3K27me3 demethylase, KDM6B, indicating that an increase in H3K27me3 levels at specific gene sites (such as those of stress‐response genes) can prolong lifespan.^[^ ^52^ ^]^ In murine hematopoietic stem cells and in *Drosophila*, the localization of the active transcription‐related H3K4me3 changes with age.^[^ ^53^, ^54^ ^]^

For oocytes, preliminary correlations between histone modification and advanced age have been reported. H3K9me2/3, H3K4me2, H3K36me2, H3K79me2, and H4K20me2 are enriched in the mouse oocyte nucleus at the GV stage and are maintained at high levels at the MII stage. While the levels of H3K9me2 and H3K4me2 do not change with age in GV oocytes, those of the other modifications at the GV and MII stages decline with age.^[^ ^24^ ^]^ The reduction in histone methylation observed in MII oocytes from older individuals may derive from GV oocytes because defects in histone methylation at the GV and MII stages are highly consistent in old oocytes.^[^ ^8^ ^]^ Histone acetylation is generally enriched in mouse oocytes at the GV stage and is eliminated at the MII stage. In oocytes from older individuals, the levels of H4K12ac and H4K16ac are reduced at the GV stage. At the MII stage, however, while normal H3K14ac and K4K16ac deacetylation occurs, H4K12ac and H4K8ac cannot be deacetylated.^[^ ^38^ ^]^ Therefore, the deacetylation ability of MII oocytes is reduced in older mice, but not completely abolished. A similar phenomenon has been observed in human oocytes. Despite these findings, it remains unclear which mechanisms or key factors affect histone modification in oocytes from older individuals.

Our findings verify that ZAR1/2 play important roles in the epigenetic aging of oocytes. Histone modifications reduced in *Zar1/2^–/–^* oocytes are also significantly reduced in oocytes from older individuals, altering their chromatin structure. MSY2 is associated with histone modifications in oocytes, suggesting that they function together. Indeed, they interact in an RNA‐dependent manner,^[^ ^18^, ^21^ ^]^ and they might affect the transcript stability of each other.^[^ ^18^, ^22^, ^55^ ^]^ MARDO and mitochondria are completely dispersed in the absence of ZAR1, partially explaining the mitochondrial disorder and mRNA instability exhibited in old oocytes.^[^ ^10^, ^21^ ^]^

**Figure 9:**
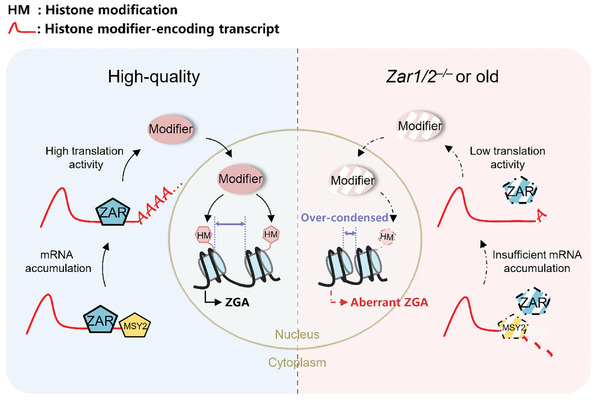
A diagram illustrating the significance of epigenetic maturation mediated by ZAR1/2. In high‐quality oocytes, the presence of sufficient levels of ZAR1/2 ensured high levels of mRNA accumulation and translation activity of MSY2 (a component of MARDO) and of multiple histone modifiers, thus contributing to appropriate histone modifications in oocytes and early embryos. In *Zar1/2^–/–^* oocytes or those from older individuals, the lack of ZAR1/2 impaired maternal mRNA accumulation and translation of MSY2 and of multiple histone modifiers, leading to disordered histone modification, increased chromatin tightness, aberrant minor ZGA, and deficient embryo development.

High‐quality oocytes contain sufficient levels of ZAR1/2 to promote the mRNA accumulation and translation activity of MARDO components such as MSY2 and of multiple histone modifiers. The assembled MARDO further maintains the transcript stability of these histone modifiers, thus contributing to appropriate histone modification in oocytes and early embryos. In *Zar1/2^–/–^* and old oocytes, insufficient ZAR1/2 levels lead to impaired maternal mRNA accumulation and insufficient translation of MSY2 and histone modifiers, followed by disruption of histone modification, increased chromatin tightness, aberrant minor ZGA, and deficient embryo development (**Figure** **9**).

Age‐associated decline in oocyte quality is complex and multifactorial, with epigenetic changes comprising a vital component of this decline. Our findings provide two potential maternal factors involved in oocyte quality and a novel perspective on nucleo‐cytoplasmic interactions, supporting future mechanistic studies of oocyte quality and epigenetic aging.

## 4 Experimental Section

### Mice

*Zar1/2* knockout mouse strains were generated via CRISPR/Cas9 system, as previously reported.^[^ ^18^ ^]^ Mice were maintained under SPF conditions in a controlled environment at 20–22 °C, with a 12/12 h light/dark cycle, 50–70% humidity, and food and water ad libitum. The experimental procedures and animal care conformed to the guidelines of the Animal Research Committee of Zhejiang University.

### Oocyte Collection and Culture

Female mice (3–4‐week‐old or 14‐month‐old) were injected with 5 IU pregnant mare serum gonadotropin (PMSG) and humanely euthanized 44 h later. Oocytes at the germinal vesicle (GV) stage were harvested in M2 medium (M7167; Sigma–Aldrich, St Louis, MO) and cultured in mini‐drops of M16 medium (M7292; Sigma–Aldrich) covered with mineral oil (M5310; Sigma‐Aldrich) at 37 °C in a 5% CO_2_ atmosphere. In some of the experiments, 2 µm milrinone was added to the culture medium to inhibit spontaneous germinal vesicle breakdown.

### Superovulation and Fertilization

Mice at 3–4 weeks of age were intraperitoneally injected with 5 IU PMSG, followed by human chorionic hormone (hCG) 44 h later. To obtain early embryos, superovulated female mice were mated with 10‐to 12‐week‐old wild type (WT) males. Successful mating was confirmed by the presence of vaginal plugs. Zygotes were harvested from the oviducts using M2 medium 28 h after hCG injection, followed by digestion using 0.3% hyaluronidase.

### Immunofluorescence

Oocytes and zygotes were fixed in 4% paraformaldehyde (PFA) in phosphate‐buffered saline (PBS) for 30 min and permeabilized in PBS containing 0.3% Triton X‐100 for 20 min. After being blocked with 1% bovine serum albumin in PBS, samples were incubated with primary antibodies for 1 h and sequentially labeled with Alexa Fluor 594‐ or 488‐conjugated secondary antibodies (Molecular Probes, Eugene, OR) and 4′,6‐diamidino‐2‐phenylindole (DAPI) for 30 min. Imaging was performed using an LSM800 confocal microscope (Zeiss, Oberkochen, Germany). The antibodies used are listed in Table S1 (Supporting Information).

### Western Blot Analysis

For Western blotting, 100 oocytes or zygotes were lysed in protein loading buffer and heated at 95 °C for 10 min. SDS‐PAGE and immunoblotting were performed according to standard procedures, using a Mini‐PROTEAN Tetra Cell System (Bio‐Rad, Hercules, CA). The antibodies used in this study are listed in Table S1 (Supporting Information).

### In Vitro mRNA Synthesis and Microinjection

To prepare the mRNAs for microinjection, the expression vectors were linearized using an appropriate restriction endonuclease. The linearized DNAs were transcribed in vitro using the Sp6 or T7 mMESSAGE mMACHINE Kit (AM1340 or 1344; Invitrogen, Thermo Fisher Scientific, Waltham, MA). The transcribed mRNAs were added by Poly(A) tails (≈200–250 bp) using the Poly(A) tailing kit (AM1350; Invitrogen), recovered via lithium chloride precipitation, and eventually resuspended in nuclease‐free water.

For microinjection, fully grown GV oocytes were collected in M2 medium with 2 µm milrinone. Microinjection was performed using an Eppendorf Transferman NK2 micromanipulator. ≈10 pL synthetic mRNAs (500 ng µL^−1^) were microinjected into the ooplasm. After injection, the oocytes were cultured in M16 medium at 37 °C at 5% CO_2_.

### *Ezh2* mRNA Fluorescence In Situ Hybridization (FISH)

The assay was performed as previously mentioned with properly modified.^[^ ^56^ ^]^ In brief, after 4–6 h in vitro culture, oocytes were washed by 0.2% BSA‐containing PBS for three times and fixed in 4% PFA‐containing PBS for 15 min, then permeabilized in PBS containing 2% Triton X‐100 for 15 min at room temperature. After rehydrated with Wash Buffer (2×SSC and 10% formamide contained) for 15 min at 37 °C, the oocytes were incubated with 1 µm Cy3‐conjugated *Ezh2* probe mix (Table S2, Supporting Information) diluted in hybridization buffer (2×SSC, 10% formamide and 10% Dextran sulfate contained) overnight at 37 °C. Then, the oocytes were washed with wash buffer for 30 min at 37 °C and then incubated with DAPI for 30 min at 37 °C. After washing with wash buffer for 30 min at 37 °C and 2×SSC for 30 min at room temperature, the oocytes were ready for imaging by LSM800.

### RNA‐Seq Library Preparation and Gene Expression Analysis

As previously described,^[^ ^18^ ^]^ ten embryos per sample were collected from the WT and *Zar1/2^–/–^* female mice for RNA‐seq. Each sample was immediately lysed with 4 µL lysis buffer (containing 0.2% Triton X‐100, RNase inhibitor, dNTPs, oligo‐dT primers, and 0.2 µL 1:1000 ERCC mRNA spike‐in dilution for mouse oocyte samples) and immediately used for cDNA synthesis using the published Smart‐seq2 method.

Raw reads were trimmed to 50 bp and mapped to the mm9 mouse genome using TopHat 2.1.1, with the default parameters. Expression levels of each gene were quantified as normalized fragments per kilobase of exons per million mapped fragments (FPKM) using Cufflinks 2.2.1. FPKM values <1 were set to 1 in subsequent analyses. Minor ZGA genes were defined as those with FC [zygote/MII] > 2.

### ATAC‐Seq

The procedure was conducted in accordance with the manufacturer's instructions for the High‐Sensitivity Open Chromatin Profile Kit 2.0 (for Illumina) (Novoprotein). In brief, 50 oocytes or zygotes per sample were transferred into 50 µL of sample lysis buffer (composed of 1 × lysis buffer, 0.3% (for growing oocytes), 0.5% (for fully grown oocytes), or 0.4% (for zygotes) NP40 and Tween 20, and 0.03% (for growing oocytes), 0.05% (for fully grown oocytes), or 0.04% (for zygotes) Digitonin), and the mixture was incubated on ice for 10 min. After lysis, 950 µL ice‐cold wash buffer (containing 1 × Lysis buffer, 0.1% Tween 20) was added to the sample, and the mixture was agitated gently and centrifuged at 500 g, 4 °C for 10 min. Then, the supernatant was discarded, and the nucleus was collected. After 40 µL of fragmentation buffer (containing 0.3 × PBS, 1 × TD buffer, 0.1% Tween 20, 0.01% Digitonin, and 4% transposome mix) was added, the fragmentation process was carried out at 37 °C for 10 min. The reaction was terminated by incubating with 5 µL stop buffer at 55 °C for 5 min and purified by beads. Afterward, the clean fragmented product was ligated with index‐containing adaptors and amplified using 1 × Hifi AmpliMix through 15–17 cycles of PCR. Finally, the libraries were again purified with beads and subjected to next‐generation sequencing on the NovaSeq X plus platform using PE150 mode.

### LACE‐Seq

Thirty oocytes per sample were harvested from WT and *Zar1/2^–/–^* mice and were irradiated twice with 400 mJ UV‐C light on ice for RNA‐binding protein–RNA crosslinking, as reported previously.^[^ ^57^ ^]^ In advance, 2 µg of ZAR1 or IgG antibody per sample was coupled with activated protein A/G magnetic beads. Cells were lysed on ice and added with the corresponding antibody‐coupled beads. Target RNAs were immunoprecipitated using beads and were fragmented using MNase. RNA dephosphorylation and first‐strand synthesis of reverse transcription were performed, and the 3′ linker and biotin‐carried T7‐RT primer were added. The first‐strand cDNA was released and re‐captured using streptavidin beads. Poly(A) tailing and pre‐amplification were performed, and the products were purified for in vitro transcription. After removing the DNA template, the remaining RNAs were purified for further reverse transcription and PCR barcoding. The LACE‐seq libraries were sequenced on an Illumina HiSeq 2500 platform by Novogene (Beijing, China).

### RIP Assay

The ribonucleoprotein immunoprecipitation (RIP) assay procedure was modified from a previously described method.^[^ ^58^ ^]^ Briefly, 800 fully grown mouse oocytes per sample, or FLAG‐ or FLAG‐hZAR1‐overexpressing 293T cells, were collected and lysed immediately in polysome lysis buffer (50 mm Tris–HCl [pH 7.4], 1% Triton X‐100, 150 mm NaCl, 5 mm ethylenediaminetetraacetic acid (EDTA), protease inhibitor cocktail, and RNase inhibitor). Of the cell lysate supernatant, 10% was used as the “input” and the remaining 90% was immunoprecipitated with protein‐A‐/‐G‐coated magnetic beads conjugated with IgG, ZAR1, or FLAG antibodies. After incubation at 4 °C for 4 h, the beads were thoroughly washed with washing buffer (50 mm Tris–HCl [pH 7.4], 0.1% Triton X‐100, 500 mm NaCl, 5 mm EDTA, protease inhibitor cocktail, and RNase inhibitor). The RNAs bound to the beads were extracted using an RNeasy Mini Kit (Qiagen, 74 106) and reverse‐transcribed using Moloney Murine Leukemia Virus (M‐MLV; Invitrogen). Relative cDNA abundance was analyzed via quantitative PCR.

### RT‐qPCR

Real‐time PCR for low cell initiation was performed with modifications to the RNA Smart‐seq protocol. Ten oocytes/embryos were collected from WT and *Zar1/2^–/–^* female mice. Each sample was lysed in 4 µL lysis buffer (containing 0.2% Triton X‐100 and 2 IU/µL RNase inhibitor, dNTPs, and oligo‐dT primers) followed by reverse transcription using SuperScript III reverse transcriptase. The product was diluted and used as a template for quantitative RT‐PCR. RT‐qPCR was performed using Power SYBR Green PCR Master Mix (Applied Biosystems, Thermo Fisher Scientific) on an ABI 7500 Real‐Time PCR system (Applied Biosystems) using the primers listed in Table S2 (Supporting Information). Relative mRNA levels were calculated following normalization to the levels of endogenous *Gapdh* mRNA (the internal control). Each experiment was performed in triplicate.

### Detection of Transcription Activity in Zygotes

To detect the transcriptional activity, zygotes were cultured in M16 medium containing 1 mm 5‐ethynyl uridine (EU) for 1 h and then fixed in 4% PFA in PBS for 30 min. EU staining was performed using a Click‐iT RNA Alexa Fluor 488 Imaging Kit (Life Technologies) according to the manufacturer's instructions.

### DNase I Sensitivity

Oocytes or zygotes were pre‐extracted for 5 min in ice‐cold solution (50 mm NaCl, 3 mm MgCl_2_, 0.5% Triton X‐100, and 300 mm sucrose, in 25 mm HEPES, pH 7.4). Then the samples were incubated with 0.1 U µL^−1^ DNase I (New England Biolabs, Ipswich, MA) for 5 min at 37 °C in the same buffer without Triton X‐100 and fixed for 10 min with 2% PFA/PBS at room temperature. A TUNEL assay was performed using the Click‐iT TUNEL Alexa Fluor Imaging Assay Kit (C10245; Life Technologies, Carlsbad, CA), according to the manufacturer's instructions.

### Statistical Analysis

The results are expressed as the mean ± SEM. Most of the experiments used at least three independent samples and were repeated at least three times. Differences between the experimental groups were evaluated using two‐tailed unpaired Student's *t*‐tests and were considered significant at *p* < 0.05 (*), *p* < 0.01 (**), or *p* < 0.001 (***). And n.s. means non‐significant.

### Ethics Statements

This study was approved by the Ethics Committee of Sir Run Run Shaw Hospital, Zhejiang University School of Medicine (No. SRRSH20220461). Animal experiments were approved by the Laboratory Animal Welfare and Ethics Committee of Zhejiang University (NO. ZJU20220410). It is confirmed that this study meets the ethical guidelines outlined in this journal's Author Guidelines, including adherence to the legal requirements of the study country.

## Conflict of Interest

The authors declare no conflict of interest.

## Supporting information



Supporting Information

## Data Availability

RNA‐seq and ATAC‐seq data have been deposited in the NCBI Gene Expression Omnibus database under the accession code GSE135787 and GSE275156, respectively.
