# Supplementary material for: ZAR1/2‐Regulated Epigenetic Modifications are Essential for Age‐Associated Oocyte Quality Maintenance and Zygotic Activation
Source: Adv Sci (Weinh). 2025 Jan 4;12(8):2410305. doi: 10.1002/advs.202410305 (PMC11848533; doi:10.1002/advs.202410305)
Supplement: Supplementary file 1 — Supporting Information [file ADVS-12-2410305-s001.docx]

**Supplementary Information**

**Supplementary Figures**


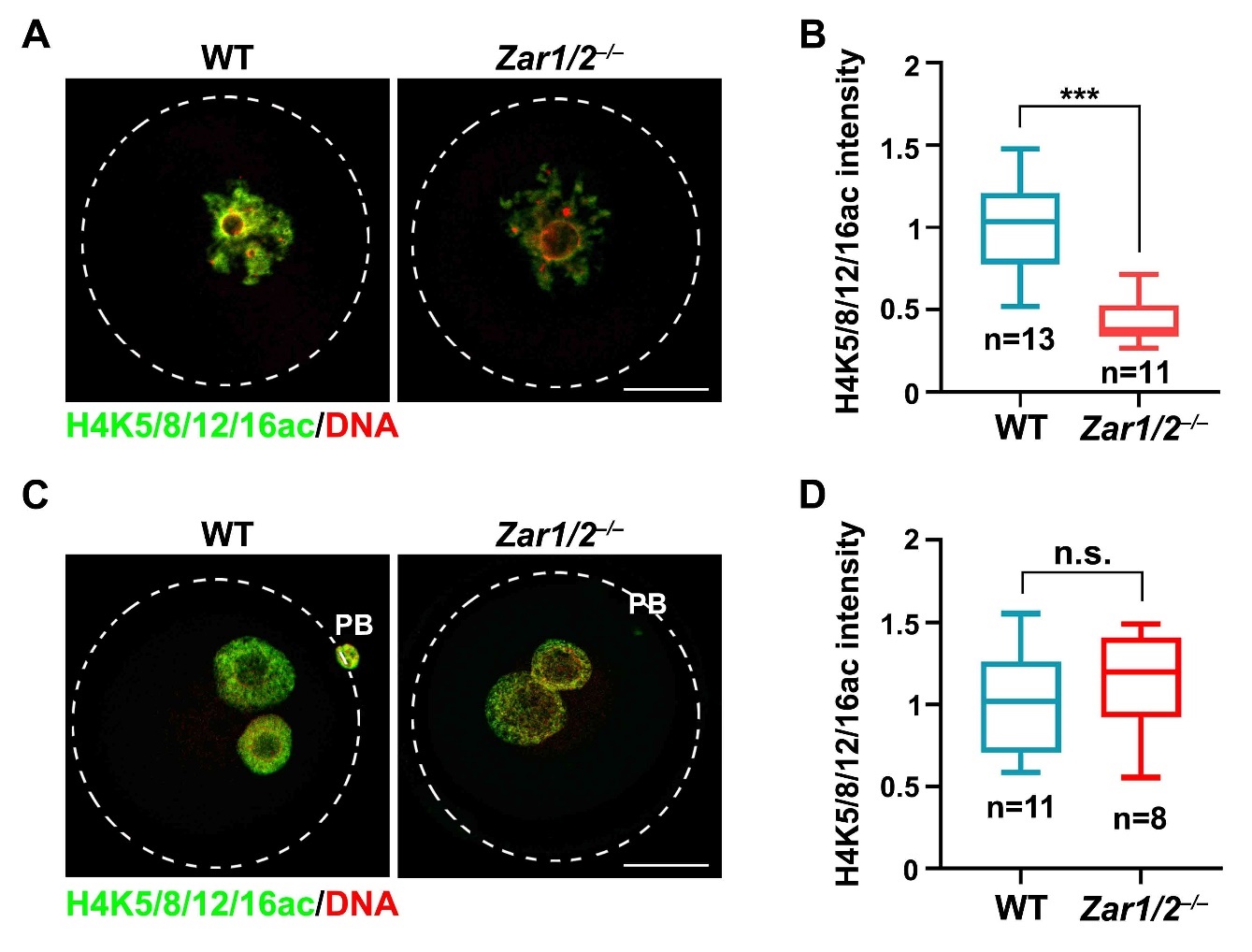


**Figure S1. Maternal ZAR1/2 contributed to proper histone modifications in mouse oocytes and zygotes. A, C:** Immunofluorescence illustrating H4K5/8/12/16ac levels in oocytes **(A)** and zygotes **(C)** from WT and *Zar1/2^–/–^* mice. Scale bar, 20 μm. **B, D:** Quantification of H4ac signal intensity of **(A)** and **(C)**, respectively. Error bars, SEM.


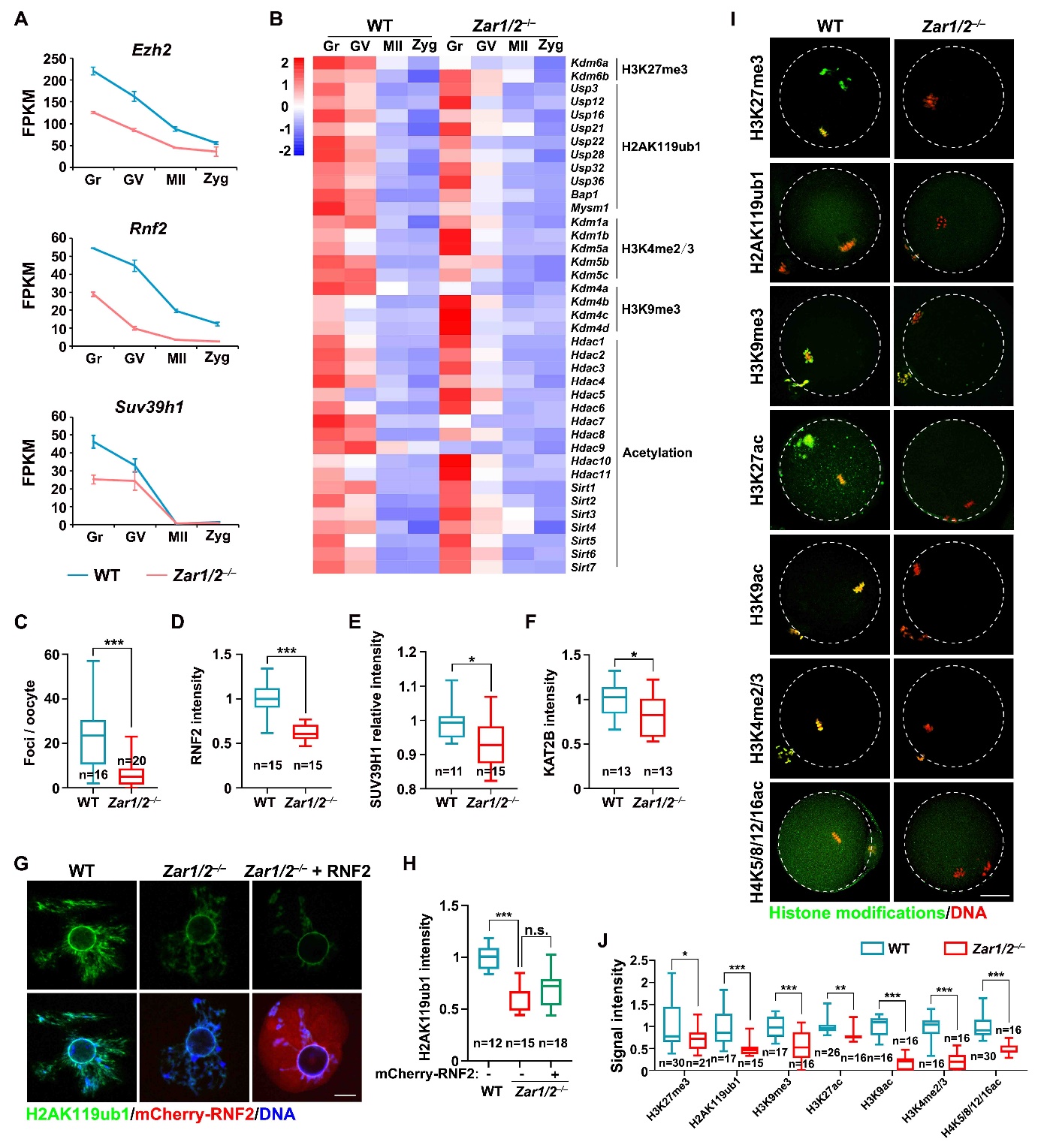


**Figure S2. ZAR1/2 deletion impaired the accumulation of transcripts and proteins that established and removed histone modifications in oocytes. A:** RNA-seq results of selected histone modifiers in oocytes and zygotes from WT and *Zar1/2^–/–^* females. **B:** Heat map illustrating the level changes of representative transcripts that removed corresponding modifications in oocytes and zygotes from WT and *Zar1/2^–/–^* females. **C:** The number of EZH2 foci of WT and *Zar1/2^–/–^* oocytes in Figure 2 **(C)**. Error bars, SEM. **D-F:** Quantification of RNF2 **(D)**, SUV39H1 **(E)** and intranuclear KAT2B **(F)** intensity in zygotes from WT and *Zar1/2^–/–^* females in Figure 2 **(D)**, **(E)** and **(F)**, respectively. Error bars, SEM. **G:** Immunofluorescence illustrating H2AK119ub1 levels in oocytes from WT and *Zar1/2^–/–^* mice, as well as *Zar1/2^–/–^* oocytes overexpressing mCherry-RNF2. Scale bar, 5 μm. **H:** Quantification of H2AK119ub1 signal intensity of **(G)**. Error bars, SEM. **I:** Immunofluorescence illustrating H3K27me3, H2AK119ub1, H3K9me3, H3K27ac, H3K9ac, H3K4me2/3 and H4K5/8/12/16ac levels of MII oocytes from WT and *Zar1/2^–/–^* mice. Scale bar, 20 μm. **J:** Quantification of histone modification levels in **(I)**. Error bars, SEM.


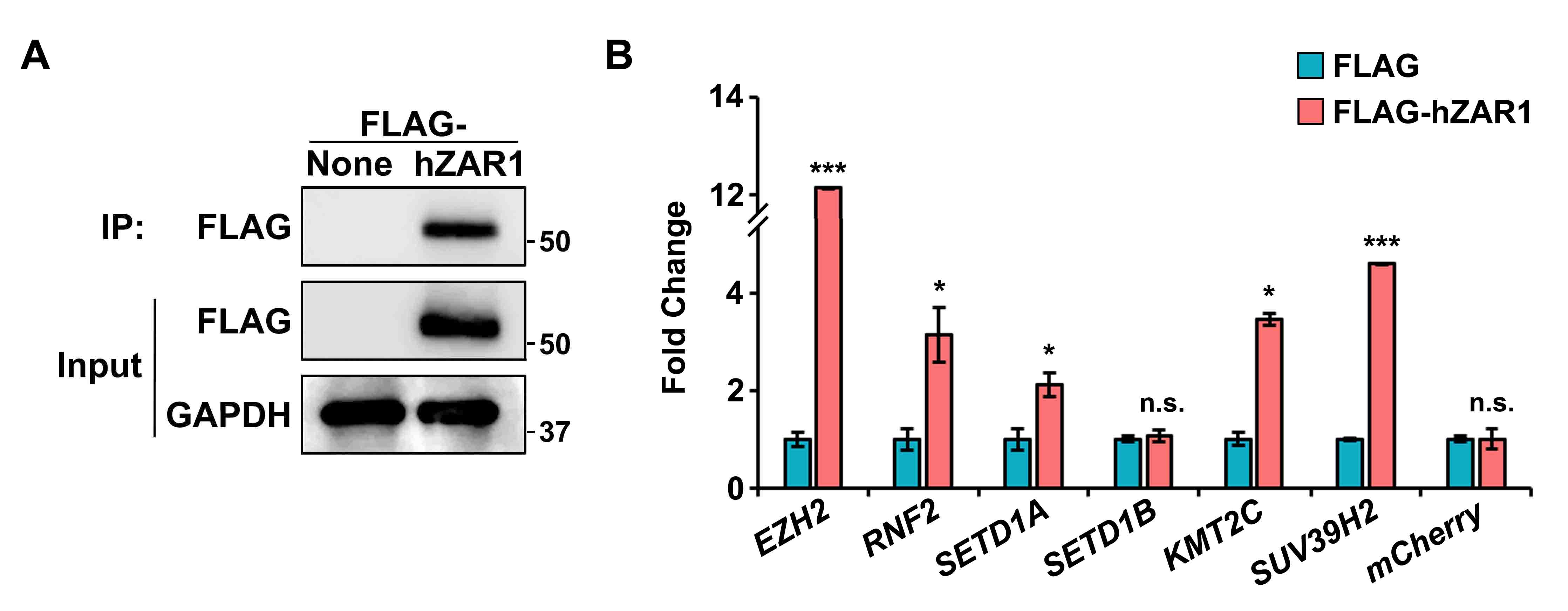


**Figure S3. Human ZAR1 interacted with histone modifier-encoding mRNAs to regulate epigenetic modifications.** **A:** Western blotting showing that FLAG-tagged hZAR1 were specifically pulled down from 293T cell lysates with the anti-FLAG antibody. **B:** RIP assay results illustrating the interactions between hZAR1 and the indicated transcripts in 293T cells. The levels of mRNAs coprecipitated with hZAR1 were detected by RT-qPCR. n = 3 biological replicates. n = 3 biological replicates. Error bars, SEM.


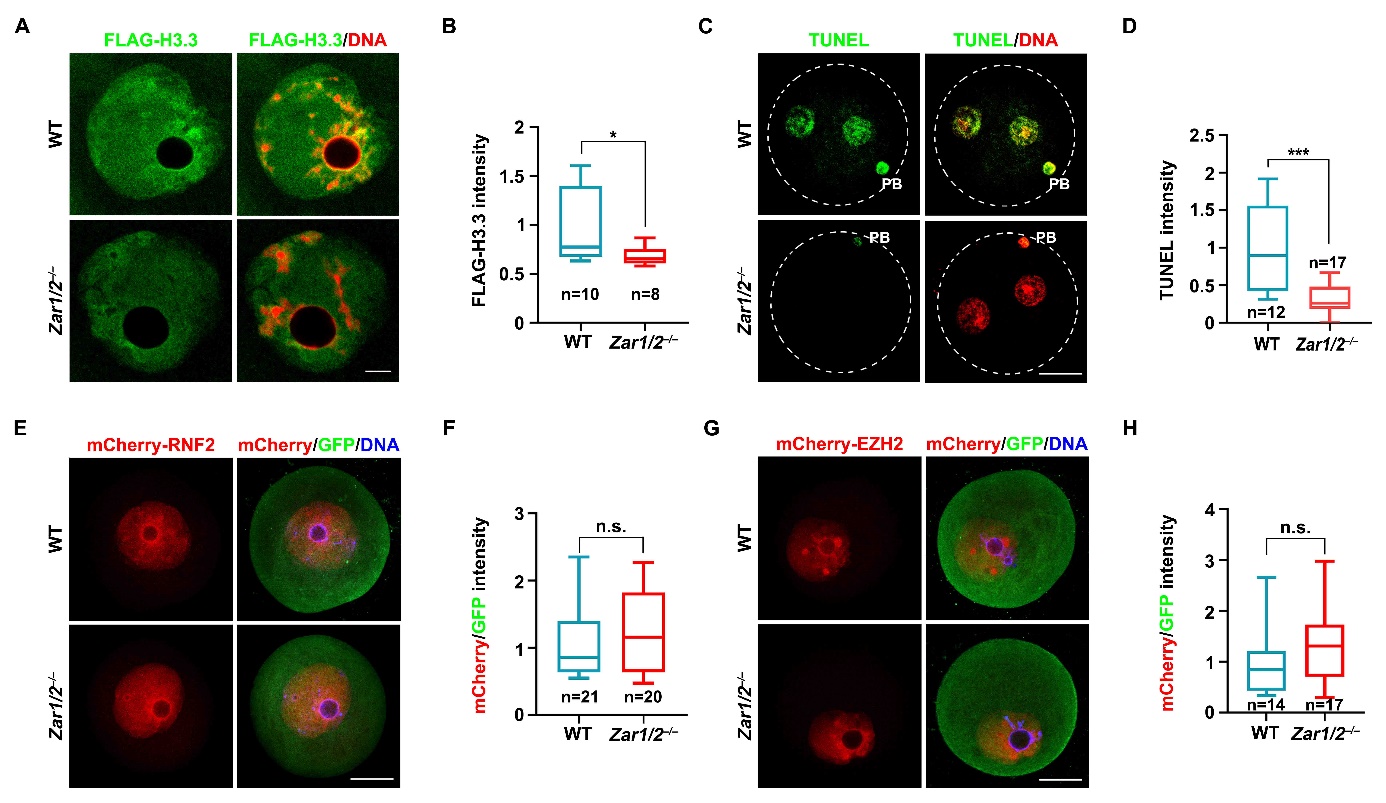


**Figure S4. Histone modification changes were caused by the reduced expression of epigenetic modifiers.** **A:** Immunofluorescence of FLAG illustrating the incorporation of FLAG-histone H3.3 in WT and *Zar1/2^–/–^* oocytes. Scale bar, 5 µm. **B:** Quantification of chromatin-incorporated FLAG-histone H3.3 signals in **(A)**. Error bars, SEM. **C:** DNase I-TUNEL assay on zygotes from WT and *Zar1/2^–/–^* females illustrating the accessibility of genomic DNA to DNase I. **D:** Quantification of TUNEL signal intensity of zygotes from WT and *Zar1/2^–/–^* females after DNase I digestion. Error bars, SEM. **E, G:** Immunofluorescence illustrating mCherry-RNF2 **(E)** and -EZH2 **(G)** levels of WT and *Zar1/2^–/–^* oocytes. *Gfp* mRNAs were co-injected as a control. **F, H:** Quantification of mCherry/GFP signal intensity of **(F)** and **(H)**, respectively. Error bars, SEM.


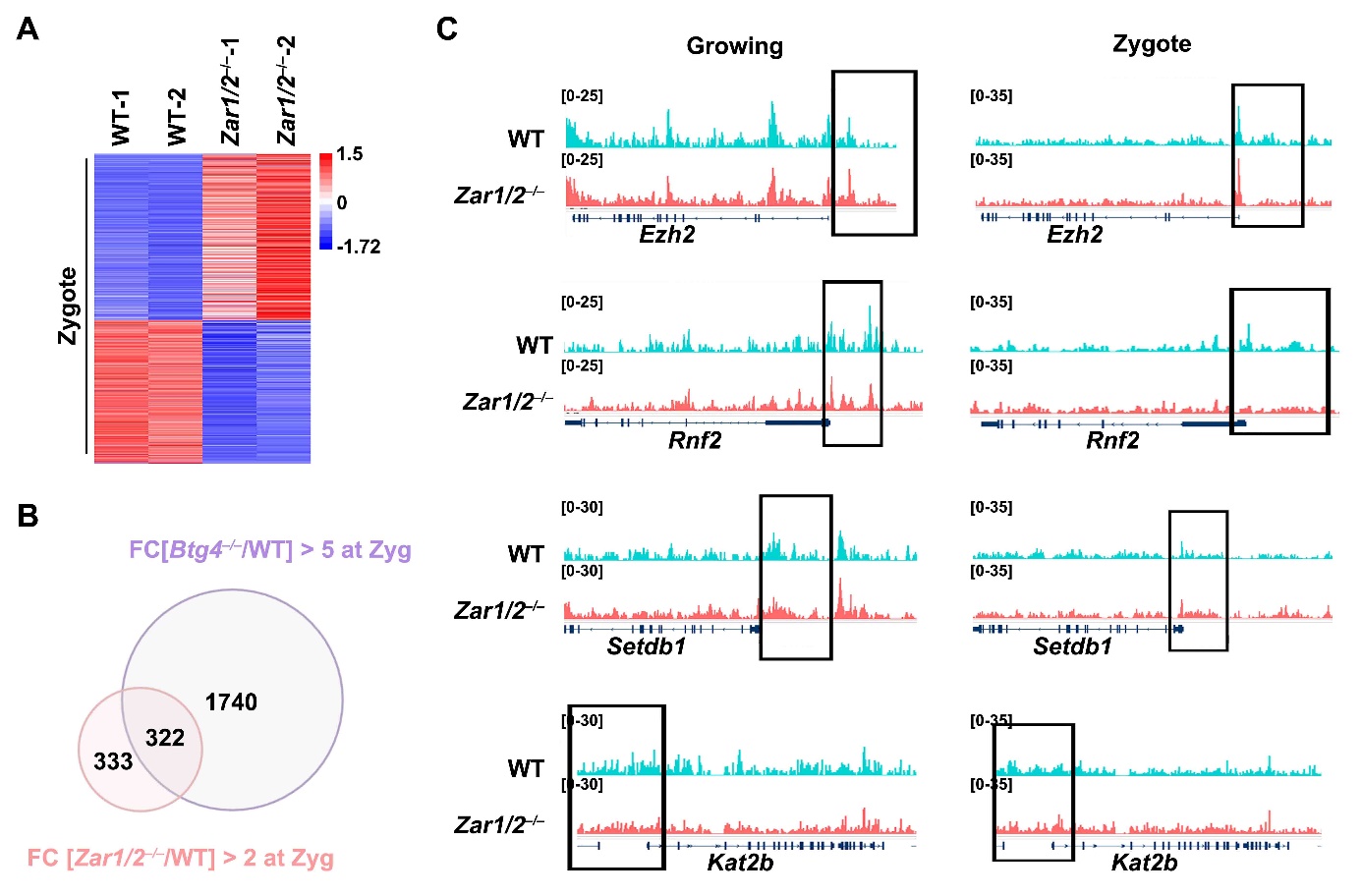


**Figure S5. The ATAC-seq enrichments of histone modifier-encoding genes were almost unaffected after ZAR1/2 deletion. A:** Heatmaps illustrating changes in transcript expression in Figure 6 **(A)**. **B:** Venn diagram illustrating the overlap of transcripts that accumulated in zygotes from *Zar1/2*- and *Btg4*-deleted females. **C:** IGV view showing ATAC-seq enrichment of histone modifier-encoding genes in growing oocytes and zygotes from WT and *Zar1/2^–/–^* females. The black frames indicated corresponding promoter regions.


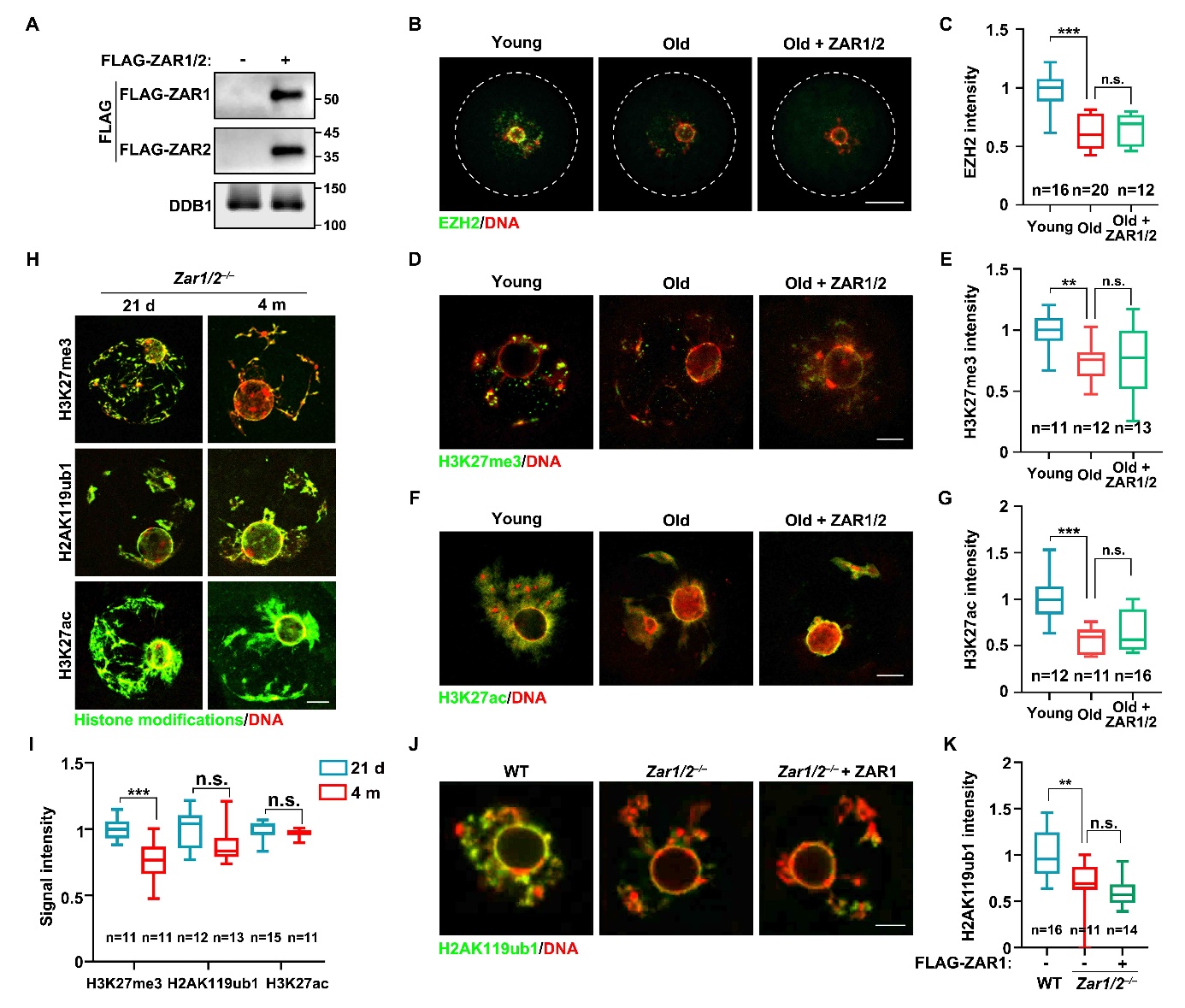


**Figure S6. Potential involvement of ZAR1/2 and histone modifications in mouse oocyte aging. A:** Western blotting results showing FLAG-ZAR1 and FLAG-ZAR2 levels in GV oocytes; DDB1 was used as a loading control. **B, D, F:** Immunofluorescence illustrating EZH2 **(B)**, H3K27me3 **(D)** and H3K27ac **(F)** levels of oocytes from younger and older mice, as well as old oocytes overexpressing FLAG-ZAR1 and FLAG-ZAR2. Scale bar, 20 µm in **(B)** and 5 µm in **(D)** and **(F)**. **C, E, G:** Quantification of modification signal intensity of **(B)**, **(D)** and **(F),** respectively. Error bars, SEM. **H:** Immunofluorescence illustrating H3K27me3, H2AK119ub1, H3K27ac levels of oocytes from 21-day-old and 4-month-old *Zar1/2^–/–^* females. Scale bar, 5 µm. **I:** Quantification of H3K27me3, H2AK119ub1 and H3K27ac levels of **(H)**. Error bars, SEM. **J:** Immunofluorescence illustrating H2AK119ub1 levels of WT and *Zar1/2^–/–^* oocytes, as well as *Zar1/2^–/–^* oocytes overexpressing FLAG-ZAR1. Scale bar, 5 µm. **K:** Quantification of H2AK119ub1 levels of **(J)**. Error bars, SEM.

**Supplementary Tables**

**Table S1. Antibody information**

| **Protein name** | **Manufacture (catalogue number)** | **Applications (working dilution)** |
| --- | --- | --- |
| H3K27me3 | Cell Signaling (9733) | IF (1:200) |
| H2AK119ub1 | Cell Signaling (8240) | IF (1:400); WB (1:1000) |
| H3K9me3 | Cell Signaling (5237) | IF (1:200); WB (1:1000) |
| H3K27ac | Abcam (ab177178) | IF (1:200) |
| H3K9ac | Abcam (ab177177) | IF (1:200) |
| H3K4me2/3 | Abcam (ab213224) | IF (1:200) |
| H4K5/8/12/16ac | Active Motif (39026) | IF (1:200) |
| EZH2 | Cell Signaling (5246) | IF (1:200); WB (1:1000) |
| RNF2 | Proteintech (16031-1-AP) | IF (1:200); WB (1:1000) |
| SUV39H1 | Abcam (ab38637) | IF (1:200) |
| KAT2B | Santa Cruz (13124) | IF (1:200) |
| CFP1 | Abcam (ab198977) | WB (1:1000) |
| ZAR1 | Made by Abcam company | WB (1:3000); IP (1:1000) |
| ZAR2 | Made by company | WB (1:5000) |
| MSY2 | Abcam (ab154829) | IF (1:500); WB (1:1000) |
| H3 | Cell Signaling (9715) | WB (1:1000) |
| DDB1 | Epitomics (3821-1) | WB (1:10000) |
| FLAG | Sigma (F3165) | IF (1:200); WB (1:1000); IP (1:200) |
| α-tubulin (11H10) | Cell Signaling (2125S) | WB (1:500) |
| GAPDH | Proteintech (60004-1-Ig) | WB (1:5000) |

**Table S2. Primer sequences**

| **Primer name** | **Target Gene** | **Application** | **Sequences (5′-3′)** |
| --- | --- | --- | --- |
| *Zar1* WT-F | *Zar1* | Genotyping of WT allele | GCTCGGCTAATCTCGGTGATGA |
| *Zar1* WT-R |  |  | GCTTTGGTGGCCTGCGGA |
| *Zar1* KO-F |  | Genotyping of KO allele | TCGGCTAATCTCGGTGATGATT |
| *Zar1* KO-R |  |  | CAGCCATCCCCGGCTTTATA |
| *Zar1*-F |  | Real-time PCR | AGAGCGCCTATGTGTGGTGT |
| *Zar1*-R |  |  | TCTCCCACACAAGTCTTGCC |
| *Zar2* GT-F | *Zar2* | Genotyping | GCTCTGGAATAAATTAAGAAACCTG |
| *Zar2* GT-R |  |  | TCCTGGCTTTCCTCGTCTTC |
| *Zar2*-F |  | Real-time PCR | TATTTCAAACAGCTCTGTAACAAGTGCC |
| *Zar2*-R |  |  | AGGAGAATTTCTTGTCTTTGCAGTGG |
| *Ezh2*-F | *Ezh2* | Real-time PCR | TGATTTTGTGGTGGATGCAACC |
| *Ezh2*-R |  |  | CTCGTTCGATGCCCACATACTTC |
| *Suz12*-F | *Suz12* | Real-time PCR | CAGTGATACCTGCTTACCTCTTCG |
| *Suz12*-R |  |  | ACAAACAGCATACAGGCATGATTC |
| *Rnf2*-F | *Rnf2* | Real-time PCR | GAGTTACAACGAACACCTCAGG |
| *Rnf2*-R |  |  | CAATCCGCGCAAAACCGATG |
| *Cbx2*-F | *Cbx2* | Real-time PCR | GGCTGGTCCTCCAAACACAA |
| *Cbx2*-R |  |  | CCCTGGGTCTCTTGCCTCT |
| *Setd1a*-F | *Setd1a* | Real-time PCR | TGCTGTCCCTCGTAGACTGG |
| *Setd1a*-R |  |  | GGCTCTTTCCGTTTTACCTTGA |
| *Setd1b*-F | *Setd1b* | Real-time PCR | TCCTCAAGCTCCGACAAGGAT |
| *Setd1b-*R |  |  | CGTCGATGTCTGAATCAATCTGG |
| *Kmt2b*-F | *Kmt2b* | Real-time PCR | GATGAGAATGGCTCGTTGTGG |
| *Kmt2b-*R |  |  | TCTATCTTGTCACACTTCCGGTA |
| *Kmt2d*-F | *Kmt2d* | Real-time PCR | GTGGCTGTTCCACACCCAG |
| *Kmt2d-*R |  |  | AGCTTGAGCTTCTCAGCATCG |
| *Suv39h1*-F | *Suv39h1* | Real-time PCR | GCAGTGTGTGCTGTAAATCTTCT |
| *Suv39h1*-R |  |  | ATACCCACGCCACTTAACCAG |
| *Suv39h2*-F | *Suv39h2* | Real-time PCR | ATCTACGAATGCAACTCAAGGTG |
| *Suv39h2*-R |  |  | CCACAGCCATTGCTAGTTCTAA |
| *Setdb1*-F | *Setdb1* | Real-time PCR | CAACTTGCACAATCACGGAAAC |
| *Setdb1*-R |  |  | CAAAGGTGACCGATATGTCTGG |
| *Ehmt2*-F | *Ehmt2* | Real-time PCR | GAAGTCGAAGCTCTAGCTGAAC |
| *Ehmt2*-R |  |  | TGAGGAACCCACACCATTCAC |
| *Kat2a*-F | *Kat2a* | Real-time PCR | AAGGCCAATGAAACCTGCAAG |
| *Kat2a*-R |  |  | CTCACAGCTACGGCACAACTC |
| *Kat2b*-F | *Kat2b* | Real-time PCR | CGGATCGCCGTGAAGAAGG |
| *Kat2b*-R |  |  | CATTGCATTTACAGGACTCCTCT |
| *P300*-F | *P300* | Real-time PCR | TTCAGCCAAGCGGCCTAAA |
| *P300*-R |  |  | CGCCACCATTGGTTAGTCCC |
| *Cbp*-F | *Cbp* | Real-time PCR | GGCTTCTCCGCGAATGACAA |
| *Cbp* -R |  |  | GTTTGGACGCAGCATCTGGA |
| *28S*-F | *28S* rRNA | Real-time PCR | CGTGTGAGTAAGATCCTCCACC |
| *28S*-R |  |  | GAGTTTACCACCCGCTTTGG |
| *hEZH2*-F | *hEZH2* | Real-time PCR | ATGAATTCATCTCAGAATACTGTGG |
| *hEZH2*-R |  |  | CTTTTGCATAGCAGTTTGGATT |
| *hRNF2*-F | *hRNF2* | Real-time PCR | GCCACTGTTGATCACTTATCCA |
| *hRNF2*-R |  |  | TCCATGGGTTTGTTCACTTTC |
| *hSETD1A*-F | *hSETD1A* | Real-time PCR | GTCAGATGGTGGCCGACATG |
| *hSETD1A*-R |  |  | CAGCTCTCTGTGCCACACAG |
| *hSETD1B*-F | *hSETD1B* | Real-time PCR | GACACCATCATCGACGCCAC |
| *hSETD1B*-R |  |  | CAGTTCTCGGAGCCACAGAGG |
| *hKMT2C*-F | *hKMT2C* | Real-time PCR | GACCATGTGATTGACGCGACG |
| *hKMT2C*-R |  |  | CATCCACTTCCGGCAGTTCAC |
| *hSUV39H1*-F | *hSUV39H1h* | Real-time PCR | GTTGTGACCCCAACCTGCAG |
| *hSUV39H1*-R |  |  | GAGGTATTTGCGGCAGGACTC |
| *hSUV39H2*-F | *hSUV39H2* | Real-time PCR | GTGGATGCGGCTCGATACG |
| *hSUV39H2*-R |  |  | GGTAACCTCTGCAAGTCACAGC |
| *mCherry*-F | *mCherry* | Real-time PCR | CCGTAATGCAGAAGAAGACCATG |
| *mCherry*-R |  |  | GCGTTCGTACTGTTCCACGATG |
| *Rpl39*-F | *Rpl39* | Real-time PCR | TGATTCGGCTTCTCGCCATGTC |
| *Rpl39*-R |  |  | CGTTCTCCTCCAGTGTCTTCTCTTAGAG |
| *Zp1*-F | *Zp1* | Real-time PCR | CCCTGAGATTGGGTCAGCG |
| *Zp1*-R |  |  | AGAGCAGTTATTCACCTCAAACC |
| *Polr2j*-F | *Polr2j* | Real-time PCR | GCCTTCGAGTCGTTCTTGCT |
| *Polr2j*-R |  |  | GCGATTTAATGATGTTCCCCAGA |
| *Cdk7*-F | *Cdk7* | Real-time PCR | ACTGTCCGGTGGAGGCATTA |
| *Cdk7*-R |  |  | CTGCTCTTTTCCGCTTTGTTG |
| *Eif4h-*F | *Eif4h* | Real-time PCR | AGAATTTGATGAGGTGGATTCCC |
| *Eif4h-*R |  |  | CCCTGTAGCCAGAGTTGAAGTC |
| *Calm2*-F | *Calm2* | Real-time PCR | ACGGGGATGGGACAATAACAA |
| *Calm2*-R |  |  | TGCTGCACTAATATAGCCATTGC |
| *Vmp1*-F | *Vmp1* | Real-time PCR | CCAGAGACGCATAGCAATGAG |
| *Vmp1*-R |  |  | GCAAGGTAATGAGTGGCTGTC |
| *Orf19*-F | *Orf19* | Real-time PCR | CGGGCCAGCTTTATGCAGT |
| *Orf19*-R |  |  | CAGCTCTACTCGTCGGGAGAT |
| *Gapdh*-F | *Gapdh* | Real-time PCR | ACACTGAGGACCAGGTTGTCTC |
| *Gapdh*-R |  |  | TACTCCTTGGAGGCCATGTAG |
| Probe 1 | *Ezh2* | FISH assay | /Cy3/GAGTACATTATAGGCACCGA/Cy3/ |
| Probe 2 |  |  | /Cy3/TCGTCGACAGAAGAGCGTAT/Cy3/ |
| Probe 3 |  |  | /Cy3/AGATGCTGGTAACACTGTGG/Cy3/ |
| Probe 4 |  |  | /Cy3/GACACCGAGAATTTGCTTCA/Cy3/ |
| Probe 5 |  |  | /Cy3/AAACATGGAGGCTTCAGCAC/Cy3/ |
| Probe 6 |  |  | /Cy3/AGGTGCTATGATACTGGACT/Cy3/ |
| Probe 7 |  |  | /Cy3/ATCACACAAGGGCACGAACT/Cy3/ |
| Probe 8 |  |  | /Cy3/CTTTGATAAAGATGCCCCAG/Cy3/ |
| Probe 9 |  |  | /Cy3/GTTGCATCCACCACAAAATC/Cy3/ |
| Probe 10 |  |  | /Cy3/CAAGGGATTTCCATTTCTCG/Cy3/ |

*h*: means human.
